# Satisfaction with remote teaching during the first semester of the COVID-19 crisis: Psychometric properties of a scale for health students

**DOI:** 10.1371/journal.pone.0250739

**Published:** 2021-04-28

**Authors:** Cristhian Pérez-Villalobos, Juan Ventura-Ventura, Camila Spormann-Romeri, Roberto Melipillán, Catherine Jara-Reyes, Ximena Paredes-Villarroel, Marcos Rojas-Pino, Marjorie Baquedano-Rodríguez, Isidora Castillo-Rabanal, Paula Parra-Ponce, Nancy Bastías-Vega, Débora Alvarado-Figueroa, Olga Matus-Betancourt

**Affiliations:** 1 Departamento de Educación Médica, Universidad de Concepción, Concepción, Chile; 2 Facultad de Salud, Universidad de Tarapacá, Arica, Chile; 3 Coordinación de Gestión Educativa en Salud, CGES, Universidad de los Lagos, Osorno, Chile; 4 Universidad del Desarrollo, Concepción, Chile; 5 Facultad de Ciencias de la Salud, Universidad de Antofagasta, Antofagasta, Chile; 6 Departamento de Ciencias de la Salud, Universidad de Aysén, Coyhaique, Chile; 7 Centro de Enseñanza y Aprendizaje, Universidad de Chile, Santiago, Chile; University of Botswana Faculty of Medicine, BOTSWANA

## Abstract

**Introduction:**

Due to the health crisis caused by the COVID-19 pandemic, 220 million college students in the world had to halt face-to-face teaching and migrate to what has been called Emergency Remote Teaching, using virtual media, but without adequate preparation. The way this has impacted the student body and its satisfaction with the training process is unknown and there are no instruments backed by specific validity and reliability studies for this teaching context. This is why this study aims to analyze the psychometric properties of the Remote Teaching Satisfaction Scale applied to Chilean health sciences students.

**Method:**

Quantitative study by means of surveys. We surveyed 1,006 health careers undergraduates chosen by convenience sampling. They came from six Chilean universities, located over a distance of 3,020 kilometers and followed 7 different careers. Women comprised the 78.53%. They answered the Remote Teaching Satisfaction Scale online to evaluate their perception of the first Emergency Remote Teaching term in 2020.

**Results:**

A descriptive analysis of the items showed a moderate to positive evaluation of the teaching. The Confirmatory Factorial Analysis showed an adequate adjustment of the theoretical four factors model to the data obtained (CFI = 0.959; TLI = 0.953; RMSEA = 0.040). Correlations among factors oscillated from r = 0.21 to r = 0.69. The measurement invariance analysis supported the Configural, Metric and a partial Scalar model. Differences were found in three of the four factors when comparing the first-year students with those of later years. Finally, the Cronbach’s α and McDonald’s ω coefficients were over 0.70.

**Discussion:**

The results display initial psychometric evidence supporting the validity and reliability of the Remote Teaching Satisfaction Scale to assess academic satisfaction in Chilean health careers students. Likewise, it is seen that first-year students show higher satisfaction levels about the implemented teaching.

## Introduction

On March 11, 2020, the World Health Organization stated that COVID-19, a disease derived from the SARS-CoV-2 virus had become a pandemic [1]. Faced with this, most of the world’s countries established quarantine and lockdown measures [2], so that at the beginning of next month, 3.4 thousand million people of over 80 countries were confined [1], with a direct and radical impact on persons’ health, mobility, freedom, work, economy, social life and education, that is still difficult to measure [1, 3].

### Effects on higher education

As part of those measures, many countries began to apply total or partial closures of their educational systems and, according to UNESCO statistics [4], the most complex moment was that of 2 April 2020, when over 1,484 million students of all education levels were affected (84.8% of the global total): 172 countries having totally closed their educational systems. In higher education, Bassett indicates that during that same month, 170 countries and communities had closed their higher education institutions, affecting over 220 million college students [3].

Since it was no longer possible to carry out face-to-face traditional teaching activities [5], universities were forced to migrate to remote teaching through online courses [1], even in those countries that had been successful in containing the pandemic such as Vietnam [6].

### Emergency remote teaching

When we refer to e-learning, we refer to that where formation activities and the delivery of materials are made on line. This includes classes, homework and assessments [7]. When well designed, it has advantages, as it enables access to education to persons who have difficulties with face-to-face training [8] or who cannot pay transport to the city of the university, or the cost of living in it [9]. But, for online teaching to work well, a well-planned course is required, an adequate preparation of the teachers to dictate it, sufficient institutional support, the creation of learning communities [8] and to guarantee the access and management of the technologic equipment for students and teachers and the connectivity to Internet [10].

Unfortunately, what was obtained after the forced closing of the institutions was not the best face of online teaching. On the contrary, what was implemented has been called Emergency Remote Teaching, characterized as presenting a fast and transitory solution to the impossibility to keep up face-to-face work due to a passing problem, and hoping to return to the prior mode after that. Its purpose is to keep up formation going even if it could deplete quality, mainly because the abrupt change makes it quite probable that the institutional resources capacity, geared to face-to-face work, would be exceeded, including the support personnel for teachers and students, support systems and the control of the teachers themselves on the courses operation. The above, without even considering that, in an emergency context such as the COVID-19 pandemic, it is probable that the community itself (students, teachers, etc.) would be affected by the health emergency itself and would not have education as its immediate priority [11].

Moreover, in the health careers case, the traditional formation structure is heavily grounded in the Flexner model which promotes learning basic knowledge before mastering more complex abilities [12], such as clinics which usually take place at the patients’ bedside training. This structure was affected, since in many cases the students were removed from clinical spaces [13]. There were fewer clinical teachers to carry out teaching, classes continuity was lost [14], and the students also saw their direct contact with patients interrupted [15].

### Effects on the teachers

A factor of no smaller importance was the effect that this had on the teachers, who were forced to migrate quickly to this new mode, without many of them having the necessary competences for online teaching or the use of technologies [1, 16]. In fact, according to a study by Watermeyer, the preparation and confidence declared by United Kingdom teachers was very heterogeneous [9].

And this becomes worse when one thinks that an online course requires six to nine months’ preparation [11]. However, in the context of the health crisis, teachers had to have their online courses ready in days or weeks; many fell back to trial and error [2] and other appealed to colleagues and social networks [9, 16]. Although it is to be expected that they will feel more comfortable the next time they give the course, it is also to be expected that the first time will be arduous, stressful and that they will be more centered in maintaining the educational service than in the students’ learning [11]. The stress of having to adapt oneself, not knowing how to do so, having to abandon old practices–even those that had been successful–and the pressure to innovate against time generate additional stressors [17].

### Effects on the students

In the students’ case, they, like the teachers, reported to be worried about bad Internet connectivity and the constraints for access to the platforms used during the Emergency Remote Teaching [16, 18]. This was not surprising. Although online teaching can facilitate access to people with scarce available time or geographically remote [9, 11], in practice millions of students and teachers do not have the conditions to actively involve themselves in the online education platforms [10].

In the case of college students, many do not have the infrastructure nor adequate connectivity in their homes, although we believe that they are better prepared for online teaching than primary or high school students, there are no major differences with the access of the latter [3].

To this is added that the crisis victims are not equally distributed in the population [19]. The students of the more vulnerable sectors face greater problems of access to the equipment and necessary connectivity, but also show more difficulties to handle the technology, generating a breach that can paradoxically increase when their university implements new technological teaching resources [10].

And finally, since all activities take place at home, online teaching can interfere with the students’ private life [20].

### Satisfaction with teaching

In spite of these adverse conditions, available studies to date document that the students report to be basically satisfied with the Emergency Remote Teaching implemented to face the COVID-19 crisis. In countries such as Vietnam [6] and Indonesia [21], students have shown more preference for this methodology than with face-to-face classes, especially in the case of first-year students. This could be due to the fact their courses are mainly theoretical and adapt better to remote teaching than higher courses [21]. While in China [22] and Poland [23] evaluation has been basically positive, although Polish students would prefer to return to face-to-face teaching.

However, some typical problems of this type of teaching are identified, such as difficulties to motivate the students, less interaction with the teachers, deficient teaching abilities, unsteadiness of the platforms [22], less social contact, loss of opportunities to train clinical abilities [5] and the difficulties of the Internet connection [21]. Besides, some medical students report fears about the pandemic affecting their specialty choice, their exploration of options or their access to letters of recommendation [24].

### Purpose of this study

To date, there are no studies about the satisfaction of college students in Latin-American, nor in Chile, a country where the first academic term normally takes place between the months of March and July and where most of the universities did not have even one week of face-to-face classes during 2020 before they closed and were forced to migrate to Emergency Remote Teaching.

Likewise, although there are instruments to measure academic satisfaction, whose validity and reliability evidence has been previously assessed in Chilean university students [25, 26], none is specifically adapted to online teaching or aspects proper to Emergency Remote Teaching.

For this reason, this study proposes a new questionnaire created by the authors of this research: the Remote Teaching Satisfaction Scale, which seeks to assess the students’ experiences in this atypical formation scenario due to the COVID-19 health crisis, from the point of view of their satisfaction with training process.

Satisfaction has been chosen as variable, because it allows to carry out an assessment of the reaction of the participants in a formation experience and is the first level to be assessed before learnings, behavior changes and results achieved [27, 28]. Although it does not replace them and must be complemented with assessments of those other elements, it is a common basis of the formation activities assessments, as it permits to explore the educational experience [29, 30] and its adequate management allows to generate a closer relationship with the students [31].

This study aims to answer the following research questions: There is evidence to support the Remote Teaching Satisfaction Scale’s valid use to assess academic satisfaction in Chilean health sciences students in the COVID-19 crisis? Are its measures reliable? And, is there measurement invariance between this scale assessment for first-year and higher year’s students?

In order to solve these questions, the research objective is to analyze the psychometric properties of the Remote Teaching Satisfaction Scale applied to Chilean health sciences students, evaluating its factorial structure as initial evidence of its construct validity, its reliability and the measurement invariance between first-years’ students and higher years’ students. We decided to compare these groups because in Chile the first group has known the university only through its computer screens as Emergency Remote Teaching activities had already started before they entered. We performed a comparison of satisfaction between both groups after the assessment of the invariance measurement.

## Method

We carried out a quantitative, non-experimental, transversal, analytic, and psychometric study.

### Participants

Six Chilean universities participated in this study, permitting a representative geographical coverage of the country: there is a straight-line distance of 3,020 kilometers between the northernmost and southernmost participating universities. Also, these universities have diverse economic students’ profiles, enrolling between 0.7% and 32.6% of students from private secondary schools, where the country’s wealthiest population studies [32].

We obtained a sample of 1,006 health careers students using a non-probabilistic convenience sampling. Of these, 78.53% were women, a percentage similar to the 73.01% of the Chilean students who started health undergraduate studies in 2020 and were women [33]. Their average age was 21.11 years (SD = 3.53), and they had an average stay in the university of 2.99 years (SD = 1.92). The students came from seven different undergraduate programs of the six universities, identified in Table 1 by number only to preserve results privacy.

**Table 1:** Description of the sampled students.

Variable	Values	n; %
Gender	Female	790; 78.53%
	Male	210; 20.87%
	Other	6; 0.60%
University	University 1	398; 39.56%
	University 2	215; 21.37%
	University 3	183; 18.19%
	University 4	80; 7.95%
	University 5	78; 7.75%
	University 6	52; 5.17%
Degree	Medical technology	110; 10.93%
	Medicine	158; 15.71%
	Midwifery	197; 19.58%
	Nursing	254; 25.25%
	Kinesiology	135; 13.41%
	Speech therapy	88; 8.75%
	Nutrition and dietetics	62; 6.16%
	No response	2; 0.20%
Years of study	First year	317; 31.51%
	Second year	158; 15.71%
	Third year	153; 15.21%
	Fourth year	163; 16.20%
	Fifth year	104; 10.34%
	Sixth year or higher	111; 11.03%

### Data collection

The students answered the Remote Teaching Satisfaction Scale, prepared by the research group as a synthesis of previous experiences such as the Questionário de Satisfação Académica (QSA, Academic Satisfaction Questionnaire) of Soares and Almeida, translated and adapted to the Chilean population [25], the Curriculum Evaluation Scale developed by Glaría et al. [34] and the Lent Academic Satisfaction Scale, validated in Chile by Vergara-Morales et al. [26]. All these instruments were validated in Chile. However, they center on the formation process conditions [25, 34] or students’ well-being [26], but not in their contribution to the students’ learning. For this reason, the Remote Teaching Satisfaction Scale is centered on the impact attributed by the students to the formation conditions on the learnings achieved. It comprises 22 items (S1 and S2 Tables), representing affirmations on the impact of different conditions of online teaching on learning, involving four factors:

**Contribution to learning**: Based on the unidimensional satisfaction scales model previously validated in university students [25, 26], the perception of teaching, evaluation and planning of teaching was incorporated as a single factor, centering on the degree in which the student perceived that these activities had favored his learning. Elements proper to the Emergency Remote Teaching were incorporated into its wording.
**Organized relationship**: Considering that uncertainty has characterized the present health crisis and that many students report being worried about the lack of certainties and information [5], the scale incorporated that factor, assessing the degree in which the student perceives that the teacher established a respectful, transparent and organized relationship with the student body. It was expressed as the conceptual opposite of the unregulated exigencies, identified as a kind of mistreatment during health training, which therefore increase uncertainty and discomfort information [35, 36].
**Context diagnosis**: This was focused on the assessment of the students’ perception about the actions of the teacher to obtain information about the personal situation of the student and the access to the means and technologies to participate actively in remote teaching, it being understood that the access to them and their mastering are aspects unevenly distributed in the population and may affect the students’ educational results [10].
**Students involvement**: It is understood that, from a constructivist viewpoint, the students must be the center of good teaching [37] actively involving the students [38], it is necessary to incorporate measurements to allow that the students themselves should evaluate how involved they are in this process. This is necessary to go beyond the focus of academic satisfaction as a mere evaluation of the service received [26].

The instrument offered seven answer alternatives in Likert format, from: 0 = Fully disagree to 6 = Fully agree.

Once the document had been prepared and before its application, we submitted it for a judgment of experts: 12 specialists in medical teaching, higher education, educational psychology, and psychometrics evaluated the content validity of its items. Next, we piloted a previous version of the scale in a convenience sample of 21 health science students from one of the participating universities who responded to a retrospective cognitive interview after it. We used those outcomes to make up the scale’s final version.

### Procedure

First, we obtained institutional approval of the six participant universities. Later on, the authorities of each of them were asked to virtually distribute the invitation to answer the anonymous online survey directly to the institutional e-mails of the students. Once the students received the e-mail, they were asked to voluntarily answer the survey by means of a hyperlink which led them directly to an informed consent form. Only those who consented went on to answer the survey. Those who refused ended the procedure at once.

The survey was applied between the third and fourth months of the first academic semester of 2020.

### Ethics

The Ethical Science Committee of the Faculty of Medicine of Universidad de Concepción, Chile, approved this study as the institution responsible for the project (No. CEC 10 2020). The other universities accepted this approval.

### Data analysis

First, a descriptive analysis of the Remote Teaching Satisfaction Scale items was performed.

In order to evaluate the factor structure of this scale, we used a confirmatory factor analysis (CFA). Robust maximum likelihood was used as the estimation method since Mardia’s kurtosis coefficient was 676.72.

The quality of the model fit was evaluated by using the following statistics: a) χ^2^, b) CFI, c) TLI, d) RMSEA, and a 90% confidence interval. A non-significant statistic χ^2^, values of 0.95 or higher for CFI and TLI, and a value of less than 0.08 for RMSEA [39, 40] were proposed as good fit criteria for the model.

To evaluate measurement invariance (MI) between first-year and higher level students, we analyzed a multi-group Confirmatory Factor Analysis sequence. Specifically, we compared three progressively restrictive models: Configural, Metric, and Scalar. For the Configural model, we specified the same pattern of free and fixed factor loadings for each group. We did not impose equality constraints on the intercepts, factor loadings, and residual variances across samples. For the Metric model, we constrained all factor loadings to be equal across groups. If factor loadings are not reasonably invariant over the groups, any comparison must be considered suspect because the latent variables themselves are not equivalent [41]. For the Scalar model, we constrained both intercepts and loadings to be equal across the two groups. A lack of invariance of item intercepts would suggest that differences over the groups cannot be only explained in terms of differences at the latent variable mean levels [41]. We should obtain Scalar invariance to ascertain that observed scores are the same across groups for identical factor scores.

To compare the fit for two nested models representing different levels in the sequence of measurement invariance, we used the Robust Likelihood Ratio Test, LRT (ΔMLR χ^2^). One important distinction between the Maximum Likelihood (ML) and MLR methods is that the traditional approach only takes into account of the difference in χ^2^ values (ΔML χ^2^). Moreover, the degrees of freedom cannot be employed for nested models using MLR because this χ^2^ difference has no χ^2^ distribution. Instead, it is necessary to employ a rescaled χ^2^-test (ΔMLR χ^2^) proposed by Yuan and Bentler [42]. A non-significant result for the ΔMLR χ^2^ test can be taken as evidence of measurement invariance. That is, the decrease in model fit associated with the more restricted model is not statistically significant.

As a second test of the measurement invariance, we analyzed the changes in the CFI and RMSEA indices. Following Chen’s recommendations for comparing two nested models, we used cut-off values of ΔCFI < 0.01 and ΔRMSEA < 0.015 for testing metric invariance and scalar invariance [43].

Finally, we evaluated the reliability of scales using Cronbach’s α. Additionally, we computed the McDonald’s ω reliability coefficient as it offers a more unbiased estimation of reliability [44].

All data analyses were conducted using Mplus 8.4.

## Results

### Descriptive data

Table 2 shows the descriptive analysis by item of the Remote Teaching Satisfaction Scale. To this end, the median was calculated, and a frequency analysis of each item was performed. To make reading of the latter easier–only for this analysis–the options were grouped as follows: 0 (Fully disagree), 1 (Mostly disagree) and 2 Disagree) within the “Disagree” category, the options 4 (Mostly Agree), 5 (Agree) and 6 (fully agree) within the “Agree” category, and option 3 (Neither agree nor disagree) in the “Neutral” category. Results show that all items have a median of 4.0 or over, this being the possible mean number of the scale, showing an assessment of the indicators that goes from moderate to positive. Individually, the better evaluated aspects were those related to the student’s involvement, where over 90% agreed that they had endeavored to understand the contents (item 18) and had fulfilled the tasks in due time (item 21). On the contrary, 12 was the worst evaluated item, where 45.8% of the students did not consider that the teachers asked about their personal situation, followed by 32.5% of students that did not believe that the times assigned for the activities were sufficient, a 30.3% of students disagree about the courses using diverse activities to achieve learning (item 3) and 30.0% did not believe that they were learning to apply course contents in an autonomous manner (item 1).

**Table 2:** Descriptive analysis of the Remote Teaching Satisfaction Scale Items.

English version items translated from original Spanish	Disagree		Neutral		Agree		Md
	n	%	n	%	n	%	
1. I am learning to apply the themes presented in the courses in an autonomous way	314	30.0	264	25.2	469	44.8	3.0
2. The activities carried out in the courses are useful to achieve the expected learnings.	232	22.2	284	27.1	531	50.7	4.0
3. Diverse activities are used in the courses to achieve the expected learnings.	317	30.3	266	25.4	464	44.3	3.0
4. The courses activities favor cooperation among the students.	277	26.4	272	26.0	499	47.6	3.0
5. Assessment activities are coherent with the purposes of the courses.	140	13.4	274	26.3	629	60.3	4.0
6. Assessment activities are an opportunity to continue learning	217	20.8	255	24.4	572	54.8	4.0
7. Feedback during the courses has helped learnings.	220	21.1	276	26.5	545	52.4	4.0
8. Online interaction opportunities help my learning.	193	18.6	237	22.8	609	58.6	4.0
9. Teachers have established a cordial relationship with the students.	49	4.7	90	8.6	902	86.6	5.0
10. The courses teachers have previously asked about the quality of our Access to the Internet.	214	20.6	152	14.6	675	64.8	4.0
11. The courses’ teachers have previously asked about our technological equipment (e.g. computers. tablet. etc.) availability.	285	27.5	167	16.1	586	56.5	4.0
12. The course teachers have asked about our personal situation.	476	45.8	211	20.3	353	33.9	3.0
13. Students have felt respected by the teachers during the courses.	118	11.4	155	14.9	765	73.7	4.0
14. Courses are motivating.	276	26.6	276	26.6	485	46.8	3.0
15. The course activities have a sequence that helps learning.	150	14.5	243	23.5	642	62.0	4.0
16. The times destined to the course activities are sufficient to achieve learnings.	337	32.5	287	27.7	412	39.8	3.0
17. Synchronic activities (in vivo) are carried out in coordinated times among courses.	64	6.2	127	12.3	845	81.6	4.0
18. I have taken pains to fully understand the themes presented in the courses.	39	3.8	50	4.8	945	91.4	5.0
19. I have asked every time I had doubts about the themes of the courses.	162	15.6	189	18.2	686	66.2	4.0
20. I have searched for additional information to understand the themes of the courses.	59	5.7	82	7.9	896	86.4	5.0
21. I have completed the tasks assigned in the courses in due time.	40	3.9	59	5.7	934	90.4	5.0
22. The platforms employed allow to satisfactorily carry out the course activities.	246	23.7	258	24.9	532	51.4	4.0

### Construct validity: Confirmatory factor analysis

**Fig 1:**
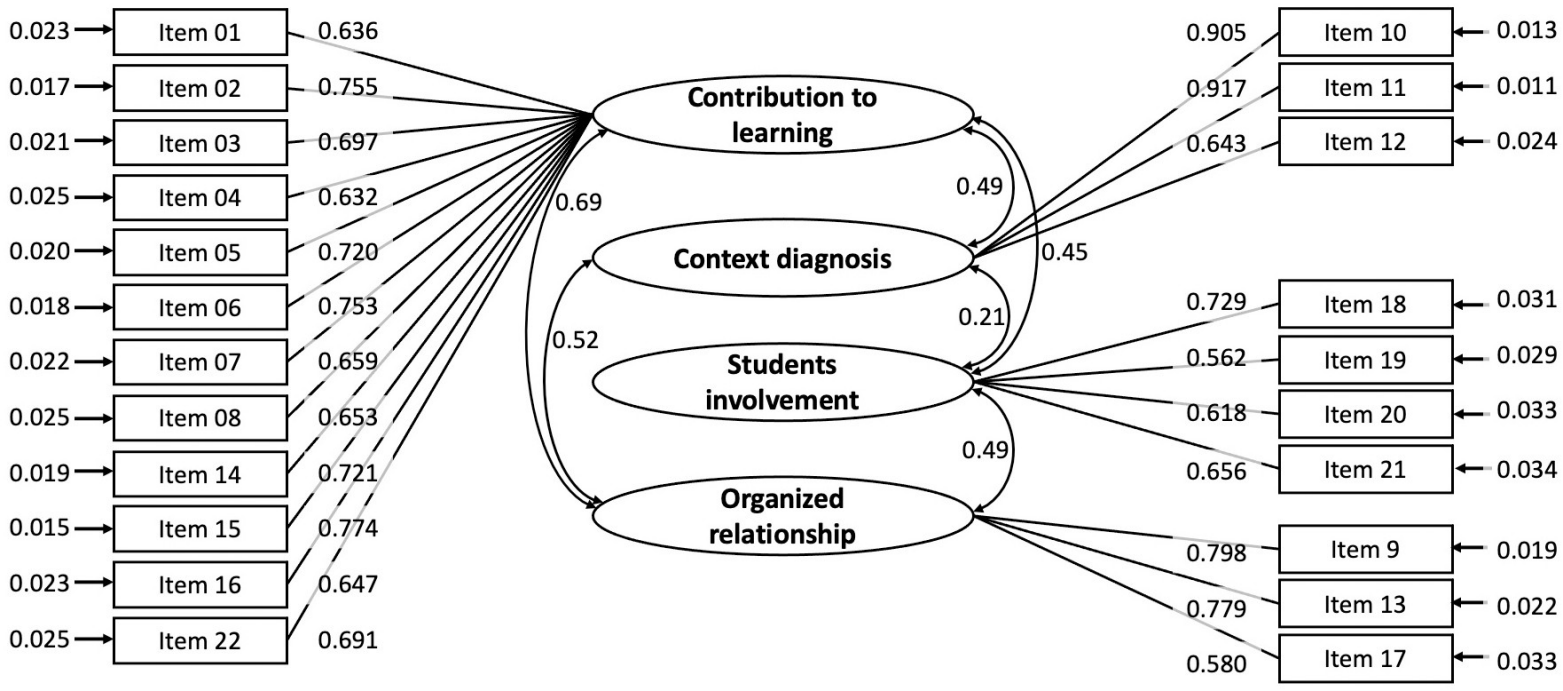
Confirmatory factor analysis of the Remote Teaching Satisfaction Scale.

Then, a CFA was performed in order to evaluate the four-factor model proposed from authors for the 22 items. Model fit results corresponded to χ^2^ (203) = 521.66, *p* < 0.001, CFI = 0.959, TLI = 0.953, RMSEA = 0.040 (90% CI: 0.035–0.044). As for the factor loadings, the absolute values in the range 0.583 to 0.918 were observed and all were statistically significant (*p* < 0.001). The correlations between the factors range from 0.22 to 0.72, Table 3. Taken together, these results provided satisfactory evidence regarding the adequacy of the four-factor model. The estimated parameters for this model are shown in Fig 1.

**Table 3:** Correlations between factors of Remote Teaching Satisfaction Scale.

	1	2	3	4
1. Contribution to learning	1.00			
2. Context diagnosis	0.49***	1.00		
3. Students involvement	0.45***	0.21**	1.00	
4. Organized relationship	0.69***	0.52***	0.49***	1.00

*N* = 1006;

*: *p* < 0.05;

**: *p* < 0.01;

***: *p* < 0.001.

### Measurement invariance

Once the factor structure for the Remote Teaching Satisfaction Scale was established, an analysis of the measurement invariance (MI) by student cohort was performed.

We first specified a four-factor configural model for freshmen and higher year students. The model fit indices of this model are shown in Table 4.

**Table 4:** Fit index of invariance models between first-year students and higher year students.

	*gl*	*χ*^*2*^	*RMSEA (IC 95%)*	*CFI*	*TLI*	Δ*χ*^*2*^	Δ*CFI*	Δ*RMSEA*
Higher year (n = 687)	203	418.080***	0.039 (0.034–0.045)	0.960	0.954	—	—	—
First-year (n = 317)	203	378.450***	0.052 (0.044–0.060)	0.929	0.920	—	—	—
Configural	410	814.458**	0.044 (0.040–0.049)	0.948	0.942	—	—	—
Metric	428	840.021***	0.044 (0.039–0.048)	0.947	0.943	24.426 (n.s.)	0.001	0.000
Scalar	446	917.106***	0.046 (0.042–0.050)	0.940	0.938	87.108***	0.007	0.002
Partial-scalar	434	852.437	0.044 (0.039–0.048)	0.947	0.943	12.580 (n.s.)	0.000	0.000

Note: n.s. denotes a non-significant Δχ2.

In the next step, we estimated the metric model and compared it with the configural model using the Likelihood Ratio Test (LRT). This procedure estimates the differences in model fit between two nested models, where model fit indices are based on the robust χ^2^. Results indicated support for metric invariance, indicating all factor loadings were invariant across the two groups.

Then, we estimated the scalar model and compared it with the metric model using the LRT. Results of this comparison showed the lack of scalar invariance, suggesting not all of the 22 intercepts tested were invariant across the two groups. We then identified a partial scalar invariant model. Based on the intercepts of the metric model and model indices of the scalar model, intercepts for items 1, 2, 3, 4, 5, 6, 13, 14, 15, 16, 17 and 18 were allowed to vary across the groups. Comparing this partial scalar model with the metric model showed partial scalar invariance. This suggests that intercepts for 8 of the 22 items were invariant between first-year and older students.

### Comparing factor means across cohorts

Finally, we compared the factor means across cohort student groups. The results indicated that the groups showed statistically significant differences for three of the four factors. Specifically, it was observed that first-year students showed higher factor means for factor 1, 3 and 4 compared to higher level students, Table 5.

**Table 5:** Factor means comparison between first-year and higher level students.

	Factor means	Standard error
Contribution to learning	0.22**	0.077
Context diagnosis	0.09	0.068
Students involvement	0.27***	0.082
Organized relationship	0.28***	0.078

### Reliability

Finally, reliability Cronbach’ α and the McDonald’ ω coefficients are shown in Table 6. Both coefficients obtained similar results for each of the four factors, all above 0.73.

**Table 6:** Reliability of Remote Teaching Satisfaction Scale.

	α	ω
Contribution to learning	0.91	0.92
Context diagnosis	0.85	0.87
Students’ involvement	0.73	0.73
Organized relationship	0.75	0.77

## Discussion

Probably very few of us in 2019 would have expected to live a 2020 year such as this, in the midst of a global health crisis that has meant a severe interruption of our daily life, with impacts on different aspects of our life and implications still difficult to foresee [1, 3].

For this reason, a constant scientific examination of our situation during 2020 is a basic and indispensable tool to make decisions, adapt to changes and prepare for what is to come, above all in the education context which has been marked by uncertainty and lack of information during this year [5].

In the health professionals’ training, we know the restrictions that this has mean for teaching, but still know little of its effects, although there already are studies about this aspect in several countries of Europe [5, 13, 23, 45, 46], North America [24] and Asia [14, 18, 21, 47], there are no evidences in this respect in Latin-America. Nor is there psychometric evidence backing up the instruments that have been employed up to date, or their suitability for an atypical formation context, such as the Emergency Remote Teaching [11].

In this study, it has been decided to convey initial evidence, choosing the academic satisfaction construct, which allows to assess the subjective experience surrounding the formation process, it being understood that this evidence must be complemented by further evaluations of the achieved learning, behavior changes, results obtained [29], and incorporate objective elements [28]. However, an adequate measurement of satisfaction that includes several criteria which should be part of a formation process evaluation must be reliable, valid, acceptable and inexpensive [28].

When analyzing each item, it is possible to find relevant evidence of this first semester functioning, where the perception of the students fluctuates between moderate and positive. This agrees with studies of countries such as Indonesia [21], Vietnam [6], China [22] and Poland [23]. In spite of the above, there are critical points. Almost half the students do not believe that their teacher has asked about their personal situation, while only a third considers that he did so. This factor is complex in a pandemic situation, where face-to-face formation activities are suspended and access to online teaching means are heterogeneous, with students having problems of access to equipment and Internet connection and others with difficulties to manage new technologies [10]. This, without mentioning that the life situation of the students was affected by the social, economic and civil upheavals of the health crisis–as everyone was. Therefore, this crisis probably made it all the more relevant that teachers should make explicit efforts to know their students’ conditions. A factor that has always been important in teaching. But it is usual to think of teaching centered more in the diagnosis of aspects as prior knowledge, learning needs, and progress of the students [48, 49], than the conditions for learning.

The analysis by items revealed another factor receiving a deficient evaluation, the adequacy of time to carry out formation activities, the learning activities diversity and the achievement of learning that would allow an autonomous application of the contents. This reveals teaching difficulties which could have appeared even before the health crisis and are an evidence of historical deficiencies in university teaching. In effect, the time assignations and variety of methodologies are two aspects to be considered in teaching scheduling, and although Chilean academicians are aware that this is the tasks that takes them more time, some studies show that they tend to orient themselves less towards student centered teaching [50, 51]. Even if the teachers recognize that–after assuming the role of student–to experiment different ways to teach helps them to learn better, when teaching the students, they tend to resort to expository strategies [52].

However–beyond the analysis by items–this study provides evidence that supports its validity and reliability to learn to what degree the scores of the Remote Teaching Satisfaction Scale deliver an adequate measurement.

Validity refers to the quality of evidence and theory to support a test measurement interpretation. One source of evidence that can support a questionnaire construct validity is the appraisal of its internal structure, assessing how the relations among their items coincide with the instrument conceptual proposal [53].

This study, starts from the premise that an adequate formation process must contribute to learning (Contribution to learning), be based on a respectful and organized relationship with the teacher (Organized relationship), have a diagnosis of the student’s conditions to accede to the learning experiences (Context diagnosis) and an active participation of the involved students (Students’ involvement). The confirming factorial analysis empirically supported this conceptual proposal of four factors, bringing evidence of the validity of its structure [53].

Firstly, the instrument allows to differentiate the contribution to learning of the formative processes which have forcibly migrated to the Emergency Remote Teaching, abandoning traditional practices both within the university campus [1, 5], due to similar constraints in all areas of knowledge, as well as the clinical contexts [13–15]. To know if the students feel that they are learning in this forced transition context, is a keystone to identify how successful have been the measures taken by the universities.

A second factor refers to the Organized relationship, that is the degree in which the teacher established a transparent and organized and therefore expectable relationship with the student. It is a central aspect, considering that it has been widely documented that the health career students–especially medical students–have been historically submitted to academic mistreatment situations, where the most frequent source is the teachers themselves. One of its manifestations is to demand over-dimensioned academic tasks that surpass the student’s available resources and its formation-level [35, 54, 55]. These sources of mistreatment generate stress [56, 57] and affect the well-being of the student [58, 59]. This is especially complex in a context where the students lack certainties about how the formation processes will continue and at what time they will be able to complete them [5]. In this sense, a respectful and planned relationship lessens uncertainty of the formation processes, something that is valuable for the students’ well-being, even out of the pandemic context.

The third factor assesses Context diagnosis, an aspect that Shulman already highlighted as a key factor of the pedagogical content knowledge that every teacher should have [60]. In effect, good teaching, besides the subject taught and pedagogic strategies requires that the teacher must be aware of the context in which it takes place. This requires that teachers should pay attention to the type of technology that students have at home, the spaces they have in which to study and their connectivity [10], aspects that also worry the students [18] and which can affect their actual access to learning opportunities. This diagnostic must be especially painstaking for the students of more vulnerable sectors, who usually have more difficulties when institutions introduce new technologies [10], so that socio-economic differences should be used as control in future research that would apply this scale.

And the last factor, students’ involvement, does not usually appear in the instruments used to assess academic satisfaction [25, 26]. However, since education is not a product to be delivered to the student, but an opportunity in which he must be actively involved to achieve the expected learnings [37, 38], this study starts based on the premise that a teaching satisfaction scale should also foster the self-evaluation of the students’ involvement.

When evaluating correlations between factors, all showed significant correlations, which is to be expected under the assumption that they all constitute indicators of an adequate educational process. But they are all under 0.70, which shows that these factors are different from one another.

Additionally, the correlations allow us to obtain information about the behavior of the construct, evidencing that the contribution to learning showed moderate to intense correlations with the other three factors, the most intense being with organized relationship. In this sense, both this last factor as well as the contribution to learning, have as basis an adequate planning of teaching. And, to the contrary, the less intense was found between the students’ involvement and context diagnosis, which could be attributed that the latter should be used as an input for teaching activities (contained in the factors Contribution to Learning and Organized Relationship) and only then can it affect the degree of participation achieved by the student.

When assessing the invariance of the measurement between students of the first-year and upper levels, it was found that the full scalar model was not supported. Due to this result, any comparisons between the two groups of students, not accounting for the non-invariance in the intercepts as mentioned above, could be biased. These results suggest the importance of performing measurement invariance tests before conducting mean comparisons across groups [61].

However, in this study the comparison of both groups was made after the MI test finding that first-year students showed higher satisfaction with the contribution to learning, students’ involvement and organized relationship in comparison with upper levels. This coincides with a previous study carried out in Indonesia, where first-year students preferred online teaching. This could be explained because the contents of first-year courses have a more theoretical emphasis, which helps their online implementation, in contrast with the more practical or integrated learnings of upper courses [21]. However, in the Chilean case, this could also be due to the fulfilling of expectations: in the case of the upper levels students, they already had university experience and expectations about the type of formation that they would receive. Furthermore, these students may resent to a greater extent the reduced opportunities to develop clinical abilities [5]. However, in Chile, the first-year students of the universities participating in the study know the emergency remote university only, so they have their school experience alone for comparison where university formats such as laboratory work or clinical formation are not present. Their present situation makes them less pressured to develop clinical abilities than older students, and they have less antecedents to critically assess the acquisition of specific abilities, such as laboratory skills.

The absence of differences in the Context diagnosis factor may be due to the fact that this activity, carried out by the teacher, implied to know the conditions of the students to accede to an online remote teaching format much more heterogeneous that the remote teaching to which each formation level was used to. In this sense, the strategies and the teacher’s degree of care to know the students’ context did not have external factors to generate variation.

Finally, the instrument showed adequate reliability coefficients, both according to Cronbach’s α which has usually been used to assess reliability, as well as McDonald’ ω which would generate less biased estimations [44]. This shows an adequate precision of the delivered measurements [62].

## Limitations

This research shows some limitations that we must consider. In the first place, although the sample represents six different universities with relevant geographic coverage of the country, we obtained them with a non-probabilistic sampling which can affect the representativity of the obtained results. In effect, the students with less access to the Internet and technologies, who can be more affected by the transition, and therefore less satisfied, may not have had access to the survey or fewer opportunities and willingness to answer it. In this sense, it represents a potential selection bias that makes it necessary to carry out future studies to deal specifically with the students’ lifestyle with less access to technologies and the adaptation experiences of those from more vulnerable sectors, ideally from a qualitative perspective.

On the other hand, we carried out this study during the first academic term of 2020, when initial efforts to migrate to the Emergency Remote Teaching had just started. This makes the study results especially valuable, as they document the first adaptation steps to this new teaching and learning way. It makes it desirable to carry out new research to assess if the student’s satisfaction improves over time. Also, to research if, with more planning time and better mastering of the technologies, the universities’ adaptation processes and the teachers become more successful than during the abrupt initial transition.

And a last limitation refers to the results, since no invariance was found in the measurements, which shows that some items are not behaving exactly in the same way in both groups. It would be important to examine this using a qualitative approach to understand what is the reason why these items are interpreted and are answered differently between the first-year students and those of higher years. This could be related to the first-year students’ participation, where they represented almost 40% of the sample with higher level students underrepresented. This was a consequence of the non-probabilistic sampling strategy we used and needs to be corrected in further studies.

## Conclusions

The results show initial psychometric evidence supporting the validity and reliability of the Remote Teaching Satisfaction Scale measures to assess the academic satisfaction of the Chilean health career students.

It also shows that the four academic satisfaction factors assessed, although different, show direct correlations among themselves, and that first-year students reported to be more satisfied with the Emergency Remote Teaching of the first term of 2020, considering its contribution to learning, the relationship with the teacher and their own involvement.

These results, although thought from the viewpoint, and in order to understand the COVID-19 health crisis effects, must not be understood as an experience restricted to it. There is consensus that this health crisis will affect life after the pandemic, will imply radical changes and people will have to reinvent themselves [1, 3].

It may be that Emergency Remote Teaching could seem to us a unique and passing experience, but this is not the first time that changes of this kind must be implemented–at least temporarily–as shown by the experiences of countries such as Afghanistan or South Africa [11]. Likewise, it is highly probable that educational institutions might face the need to interrupt their face-to-face teaching in future due to new social, climate emergencies, etc. [11], therefore disaster planning must be incorporated to the strategic plans of university organizations [19].

In this sense, although the Remote Teaching Satisfaction Scale shows adequate functioning conditions to carry out diagnostics during the present crisis, it is a tool that can be useful to assess students’ satisfaction in any remote teaching experience–not necessarily emergencies–and can be viewed as an instrument to be employed in future crises.

## Supporting information

S1 Table Escala de satisfacción con la docencia a distancia (versión original en Español). (PDF)

S2 Table Remote Teaching Satisfaction Scale (proposed English version). (PDF)
