# Supplementary material for: Satisfaction with remote teaching during the first semester of the COVID-19 crisis: Psychometric properties of a scale for health students
Source: PLoS One. 2021 Apr 28;16(4):e0250739. doi: 10.1371/journal.pone.0250739 (PMC8081238; doi:10.1371/journal.pone.0250739)
Supplement: S1 Table — (PDF) [file pone.0250739.s001.pdf]

S1 Table. Table. Escala de Satisfacción con la Docencia a Distancia (Versión original en español)

Por favor, indique la opción que mejor describa su grado de satisfacción con el proceso de formación desarrollado en línea durante este primer trimestre de 2020.

	Totalmente en desacuerdo	Muy en desacuerdo	En desacuerdo	Ni de acuerdo ni en desacuerdo	De acuerdo	Muy de acuerdo	Totalmente de acuerdo
1. Estoy aprendiendo a aplicar las temáticas abordadas en las asignaturas de forma autónoma.	0	1	2	3	4	5	6
2. Las actividades realizadas en las asignaturas son útiles para lograr los aprendizajes esperados.	0	1	2	3	4	5	6
3. En las asignaturas se están empleando diversas actividades para alcanzar los aprendizajes esperados.	0	1	2	3	4	5	6
4. Las actividades de las asignaturas favorecen la cooperación entre los estudiantes.	0	1	2	3	4	5	6
5. Las actividades de evaluación son coherentes con los propósitos de las asignaturas.	0	1	2	3	4	5	6
6. Las actividades de evaluación son una oportunidad para seguir aprendiendo.	0	1	2	3	4	5	6
7. La retroalimentación durante las asignaturas ha favorecido los aprendizajes.	0	1	2	3	4	5	6
8. Las instancias de interacción en línea favorecen mi aprendizaje.	0	1	2	3	4	5	6
9. Los docentes han establecido una relación cordial con los estudiantes.	0	1	2	3	4	5	6

10. Los docentes de las asignaturas nos han preguntado previamente por la calidad de nuestro acceso a internet.	0	1	2	3	4	5	6
11. Los docentes de las asignaturas nos han preguntado previamente por nuestra disponibilidad de equipos tecnológicos (p.e. computadores. tablet. etc.).	0	1	2	3	4	5	6
12. Los docentes de las asignaturas nos han preguntado por nuestra situación personal.	0	1	2	3	4	5	6
13. Los estudiantes se han sentido respetados por los docentes durante las asignaturas.	0	1	2	3	4	5	6
14. Las asignaturas son motivadoras.	0	1	2	3	4	5	6
15. Las actividades de las asignaturas tienen una secuencia que favorece los aprendizajes.	0	1	2	3	4	5	6
16. Los tiempos destinados a las actividades de las asignaturas son suficientes para lograr aprendizajes.	0	1	2	3	4	5	6
17. Las actividades sincrónicas (en vivo) se realizan en horarios coordinados entre las asignaturas.	0	1	2	3	4	5	6
18. Me he esforzado por comprender cabalmente las temáticas abordadas en las asignaturas.	0	1	2	3	4	5	6
19. He preguntado cada vez que tuve dudas sobre las temáticas de las asignaturas.	0	1	2	3	4	5	6
20. He buscado información adicional para comprender los contenidos de las asignaturas.	0	1	2	3	4	5	6

21. He cumplido oportunamente los tareas asignadas en las asignaturas.	0	1	2	3	4	5	6
22. Las plataformas empleadas permiten realizar satisfactoriamente las actividades de las asignaturas.	0	1	2	3	4	5	6
