# Supplementary material for: Satisfaction with remote teaching during the first semester of the COVID-19 crisis: Psychometric properties of a scale for health students
Source: PLoS One. 2021 Apr 28;16(4):e0250739. doi: 10.1371/journal.pone.0250739 (PMC8081238; doi:10.1371/journal.pone.0250739)
Supplement: S2 Table — (PDF) [file pone.0250739.s002.pdf]

S2 Table. Remote Teaching Satisfaction Scale (Proposed English version)

Please indicate the choice than better describe how satisfied you felt with the training process developed online during this first term of 2020.							
	Fully disagree	Strongly disagree	Disagree	Neither agree nor disagree	Agree	Strongly agree	Fully agree
1. I am learning to apply the themes presented in the courses in an autonomous way	0	1	2	3	4	5	6
2. The activities carried out in the courses are useful to achieve the expected learnings.	0	1	2	3	4	5	6
3. Diverse activities are used in the courses to achieve the expected learnings.	0	1	2	3	4	5	6
4. The courses activities favor cooperation among the students.	0	1	2	3	4	5	6
5. Assessment activities are coherent with the purposes of the courses.	0	1	2	3	4	5	6
6. Assessment activities are an opportunity to continue learning	0	1	2	3	4	5	6
7. Feedback during the courses has helped learnings.	0	1	2	3	4	5	6
8. Online interaction opportunities help my learning.	0	1	2	3	4	5	6
9. Teachers have established a cordial relationship with the students.	0	1	2	3	4	5	6
10. The courses teachers have previously asked about the quality of our Access to the Internet.	0	1	2	3	4	5	6
11. The courses' teachers have previously asked about our technological equipment (e.g. computers. tablet. etc.) availability.	0	1	2	3	4	5	6
12. The course teachers have asked about our personal situation.	0	1	2	3	4	5	6

13. Students have felt respected by the teachers during the courses.	0	1	2	3	4	5	6
14. Courses are motivating.	0	1	2	3	4	5	6
15. The course activities have a sequence that helps learning.	0	1	2	3	4	5	6
16. The times destined to the course activities are sufficient to achieve learnings.	0	1	2	3	4	5	6
17. Synchronic activities (in vivo) are carried out in coordinated times among courses.	0	1	2	3	4	5	6
18. I have taken pains to fully understand the themes presented in the courses.	0	1	2	3	4	5	6
19. I have asked every time I had doubts about the themes of the courses.	0	1	2	3	4	5	6
20. I have searched for additional information to understand the themes of the courses.	0	1	2	3	4	5	6
21. I have completed the tasks assigned in the courses in due time.	0	1	2	3	4	5	6
22. The platforms employed allow to satisfactorily carry out the course activities.	0	1	2	3	4	5	6
